# Lipin-1 Contributes to IL-4 Mediated Macrophage Polarization

**DOI:** 10.3389/fimmu.2020.00787

**Published:** 2020-05-05

**Authors:** Sunitha Chandran, Robert M. Schilke, Cassidy M. R. Blackburn, Aila Yurochko, Rusella Mirza, Rona S. Scott, Brian N. Finck, Matthew D. Woolard

**Affiliations:** ^1^Department of Microbiology and Immunology, Louisiana State University Health Sciences Center, Shreveport, LA, United States; ^2^Department of Pathology and Translational Pathobiology, Louisiana State University Health Sciences Center, Shreveport, LA, United States; ^3^Division of Geriatrics and Nutritional Science, Washington University School of Medicine, St. Louis, MO, United States

**Keywords:** lipin-1, macrophage, polarization, wound healing, transcriptional coregulator

## Abstract

Macrophage responses contribute to a diverse array of pathologies ranging from infectious disease to sterile inflammation. Polarization of macrophages determines their cellular function within biological processes. Lipin-1 is a phosphatidic acid phosphatase in which its enzymatic activity contributes to macrophage pro-inflammatory responses. Lipin-1 also possesses transcriptional co-regulator activity and whether this activity is required for macrophage polarization is unknown. Using mice that lack only lipin-1 enzymatic activity or both enzymatic and transcriptional coregulator activities from myeloid cells, we investigated the contribution of lipin-1 transcriptional co-regulator function toward macrophage wound healing polarization. Macrophages lacking both lipin-1 activities did not elicit IL-4 mediated gene expression to levels seen in either wild-type or lipin-1 enzymatically deficient macrophages. Furthermore, mice lacking myeloid-associated lipin-1 have impaired full thickness excisional wound healing compared to wild-type mice or mice only lacking lipin-1 enzymatic activity from myeloid cell. Our study provides evidence that lipin-1 transcriptional co-regulatory activity contributes to macrophage polarization and influences wound healing *in vivo*.

## Introduction

Macrophages are innate immune cells that mediate tissue homeostasis by polarizing into unique phenotypes that range from pro-inflammatory to wound healing. Macrophage cellular responses restore normal tissue function. Defects in macrophage polarization can influence numerous disease pathologies including infectious disease, atherosclerosis, tumor growth, and impaired wound closure. Activation of macrophages via pattern recognition receptors (i.e., Toll like receptors) or through pro-inflammatory cytokine receptors (i.e., IFN-γ or TNF-α receptor) lead to pro-inflammatory activities (1). Conversely, IL-4, IL-10, IL-13, or TGF-β stimulation of macrophages promotes wound healing activities. The binding of these cytokines to their respective receptors leads to the activation and inhibition of numerous transcriptions factors that promote polarization of macrophages to a wound healing state. Most critical to wound healing polarization is the peroxisome proliferator-activated receptors (PPAR) family of transcription factors (2). PPAR activation via ligand binding and association with co-activators leads to both trans-repressive and transactivating activity (3). In macrophages, PPARs can transrepress NF-κB and STAT1 at the promoters of pro-inflammatory cytokines such as TNF-α (4). PPARs also promote the expression of genes associated with both lipid catabolism (2) and macrophage wound-healing activity (5).

Lipin-1 belongs to the evolutionarily conserved three-member lipin family (lipin-1, -2, and -3) in mammals. Lipins enzymatically convert phosphatidate into diacylglycerol via dephosphorylation. Among lipin family proteins, lipin-1 exhibits the highest phosphatidate-specific phosphohydrolase activity (6). We and others have shown that expression of a hypomorphic lipin-1 protein that lacks enzymatic activity attenuates pro-inflammatory macrophage responses by regulating glycerolipid synthesis (7–9). Lipin-1 enzymatic activity within macrophages contributes to disease pathogenesis of atherosclerosis, colitis, colon cancer, and LPS-induced inflammation [reviewed in (10)]. The overarching mechanism is likely due to lipin-1-mediated diacylglycerol production leading to protein kinase C and AP-1 transcription factor activation driving pro-inflammatory macrophage activities (7–9). In addition to acting as a lipid phosphatase, lipin-1 also independently acts as a transcriptional co-regulator by interacting with various DNA-bound transcription factors. It is unknown if lipin-1 transcriptional co-regulator activity is involved in regulating wound healing activity in macrophages. However, lipin-1 augments PPAR activity to promote adipogenesis in adipocytes and promotes beta-oxidation while suppressing very low-density lipoprotein production in hepatocytes (11–15). Lipin-1 also represses the activity of SREBP1, SREBP2, and NFAT4c by inhibiting the binding of these transcription factors to their respective promoters in hepatocytes (16, 17). SREBP1, SREBP2, and NFAT4c have been identified to contribute to promotion of macrophage pro-inflammatory responses and inhibition of wound healing macrophage polarization (18–20). These studies suggest to us that in macrophages the potential of lipin-1 transcriptional co-regulatory activity promoting PPARs and inhibiting SREBPs and NFAT4c might be important for the polarization of macrophages to a wound healing state. Our data provides evidence that lipin-1 transcriptional co-regulator activity contributes to IL-4 mediated macrophage wound healing function.

## Materials and Methods

### Animals

All animal studies were approved by the LSU Health Sciences Center-Shreveport institutional animal care and use committee. All animals were cared for according to the National Institute of Health guidelines for the care and use of laboratory animals. After wounding, all mice were housed in individual filter-topped sterile cages, provided with sterile water and food *ad libitum*.

All animals used in this study were 8 to 10-week-old mice. Mice lacking lipin-1 enzymatic activity from myeloid cells (lipin-1^mEnzy^KO) were generated as previously reported (9). Briefly, mice with exons 3 and 4 of the *Lpin1* gene flanked by LoxP sites (genetic background: C57BL/6J and SV129; generously provided by Brian Finck and Roman Chrast) were crossed with C57BL/6J *LysM*-Cre transgenic mice purchased from Jackson Laboratory (Bar Harbor, ME, United States). Exon 3 encodes the translational start site of lipin-1; however, deletion of this exon led to enforcement of an alternative start site causing expression of a truncated lipin-1 protein lacking 115 amino acids (21). The truncated protein lacks phosphatidic acid phosphohydrolase activity but retains cotranscriptional regulatory function. Mice fully lacking lipin-1 from myeloid cells (lipin-1^m^KO) were generated by crossing mice with exon 7 of the *Lpin1* gene flanked by LoxP sites [genetic background: C57BL/6J and SV129; generously provided by Brian Finck (22)] with C57BL/6J *LysM*-Cre transgenic mice purchased from Jackson Laboratory (Bar Harbor, ME). Deletion of exon 7 leads to frameshift, premature stop codon insertion, and a complete loss of lipin-1 protein (22). Age matched lipin-1^flox/flox^ littermate mice were used as controls.

### Excisional Wound Healing Model

Mice were anesthetized by 3% isoflurane (NDC, 14043-704-06) and clippers were used to remove hair from the dorsum. Exposed skin was disinfected with chlorohexidine swabs. Dorsal skin was folded, raised cranially, and mice were laterally positioned. Symmetric full thickness wounds were created using a sterile 5mm biopsy punch (Integra) (23). Gross images were taken and percentage of wound closure was assessed using a digital caliper at 0, 2, 5, 7, 9, 12, and 14-days post-wounding and expressed as [(area of original wound – area of current wound)/area of original wound]x100. After initial wounding, analgesic cream was applied to wounds (Aspercreme, Cattem, 0078940). Mice were routinely monitored for weight loss or any other type of distress until the end of the study.

### Generation of Bone Marrow-Derived Macrophages

Bone marrow-derived macrophages (BMDMs) were generated from lipin-1^mEnzy^KO, lipin-1^m^KO and littermate control mice as previously described (24). Briefly, femurs were excised under sterile conditions and flushed with Dulbecco’s modified Eagle’s Knock out medium (DMEM; Gibco, 10829) supplemented with 10% fetal bovine serum (Atlanta biologicals, S11150), 2 mM L-glutamax (Gibco -35050-061), 100 U/ml penicillin-streptomycin (Cell Gro, 30-604-CI), 1 mM sodium pyruvate (Cell gro, 25-060-CI), and 0.2% sodium bicarbonate (Quality biological, 118-085-721). Red blood cells were lysed using ammonium chloride-potassium carbonate (0.15 M NH_4_Cl, 10 mM KHCO_3_, 0.1 mM NA_2_EDTA, adjusted to pH 7.2 and filter sterilized in 0.22 μm filter) lysis (ACK) followed by PBS wash. Isolated cells were incubated in sterile petri dishes for 7 to 10 days in BMDM differentiation medium – DMEM KO (Gibco10829) supplemented with 30% L-cell conditioned medium, 20% fetal bovine serum [Atlanta biologicals, S11150) 2 mM L-glutamax (Gibco -35050-061), 100 U/ml penicillin-streptomycin (Cell Gro, 30-604-CI), 1mM sodium pyruvate (Cell Gro, 25-060-CI)], and 0.2% sodium bicarbonate (Quality biological, 118-085-721) at 37°C and 5% CO_2_. Once cells were 80% confluent, they were collected using 11 mM EDTA, pH 7.6 treatment. 10^6^ cells were seeded for RNA extraction and 5 × 10^5^ cells for protein isolation and flow cytometry analysis. After 4 h of seeding, cells were treated with 0 or 20 ng/ml IL-4 (R&D Biosystems, 404-ML-050) for various times.

### Flow Cytometry

#### Interleukin-4 Receptor Staining

Bone marrow-derived macrophages were incubated with CD16/CD32 (e-Bioscience, 14-0161-86) for 20 min. BMDMs were then incubated with PECy7 conjugated anti-CD11b (e-Bioscience, 25-0112-81, clone M1/70), and PE conjugated anti-IL-4R (Biolegend, 144803) for 30 min in the dark. Cells were then fixed with 4% formaldehyde and analyzed using BD LSRII (San Jose, CA, United States).

#### Immune Composition Staining

Spleens were homogenized in FACS wash buffer (1% bovine serum albumin, 1 mM EDTA, and 0.1% sodium azide in phosphate buffered saline) followed by centrifugation at 300 × *g* for 5 min. The supernatant was decanted and splenocytes were dislodged in 3 ml of ACK lysis buffer. Splenocytes were incubated on ice for 5 min. Splenocytes were washed in FACS wash buffer then centrifuged. The pellet was re-suspended in FACS wash buffer and strained with a 40 μm cell strainer (Falcon, 352340), and counted. Splenocytes were adjusted to 1 × 10^6^ cells/mL in RPMI. Blood was collected in EDTA coated tubes. 100 μl of blood was lysed in 3 mls of ACK lysis buffer and then washed with FACS wash buffer. The entire sample of blood cells were stained. Splenocytes and blood cells were incubated with anti-CD16/CD32 (e-Bioscience, 14-0161-86) for 20 min. After blocking, cells were stained with a cocktail of antibodies: AF700 conjugated anti-CD45.2 (Biolegend,109821,clone104), BV605 conjugated anti-CD3 (Biolegend,100237,clone17A2), BV786 conjugated anti-CD11c (BD Biosciences,563735,cloneHL3), PECy7 conjugated anti-CD11b (eBioscience, 25-0112-81, clone M1/70), PEe610 conjugated anti-CD19 (eBioscience,61-0193-80,clone eBio1D3), FITC conjugated anti-Ly6G (BD Biosciences,551460,clone1A8), PE conjugated anti-Ly6C (eBioscience,12-5932-80,clon eHK1.4) and APC-Cy7 conjugated anti-CD115 (Biolegend,135532,cloneAFS 98). Appropriate F Minus One Controls were used to correct background and exclude spectral overlap staining. Compensation control (Comp Bead, Invitrogen, 01-2222-42) were used. Flow cytometry analysis was performed using BD LSRII (San Jose, CA). Data analysis was done using FCS express (*Denovo* Software) and NovoExpress (AceaBio).

### Wound Staining

Quantification of macrophage phenotypes within the wound was performed as previously described (25). Briefly dorsal skin was carefully removed from the euthanized mice and placed onto filter paper. 10 mm x 10 mm tissue specimen including the wounded area and adjacent tissue was made. Subcutaneous fat and muscle were removed from the tissue and wound was minced into 4–5 smaller pieces. Tissue was further digested in Dispase II enzyme cocktail (2 mg/mL, Thermo Fisher Scientific 17105-041 and 0.1-mg/mL DNase I Roche, cat. #10104159001) in a volume of 700 μL DMEM (Gibco10829) media and incubated in a shaker at 1,400 rpm, 37°C for 2 h. After incubation, undigested debris was removed by filtering the sample through 70 μm strainer. Add 500 μL of cold FACS wash buffer to the side of strainer to wash off the remaining cells into the collection tube. Centrifuge at 4°C for 5 min at 400 × *g*. Remove the supernatant and resuspend the cell pellet in 200 μL cold FACS buffer. 0.5 × 10^6^ cells were then stained first for dead cells using Invitrogen aqua live/dead stain (Thermo Fisher Scientific L34965). After live/dead staining, cells were stained with anti-CD16/CD32 (e-Bioscience, 14-0161-86) for 20 min. After blocking, cells were stained with a cocktail of antibodies: AF700 conjugated anti-CD45.2 (Biolegend,109821,clone104), PECy7 conjugated anti-CD11b (eBioscience, 25-0112-81, clone M1/70), FITC conjugated anti-Ly6G (BD Biosciences,551460,clone1A8), PE-Cy5 conjugated anti-F4/80 (Invitrogen, 15-4801-80, clone BM8), and AF647 conjugated anti-CD206 (Biolegend, 141711, clone C068C2). Appropriate F Minus One Controls were used to correct background and exclude spectral overlap staining. Compensation control (Comp Bead, Invitrogen, 01-2222-42) were used. Flow cytometry analysis was performed using Novocyte Quanteon (Aceo Bio). Data analysis was done using FCS express (Denovo Software) and NovoExpress (AceaBio).

### Western Blot

Cells were lysed in 1× NuPage LDS sample buffer [containing 100 mM dithiothreitol (DTT; Life Technologies), 1× protease inhibitor cocktail (Thermo Fisher Scientific), 1× phosphatase inhibitor cocktail 2 (Sigma Aldrich), and 1× phosphatase inhibitor cocktail 3 (Sigma Aldrich)]. Protein concentration was determined by Peirce^TM^ 660 nm Protein Assay (Thermo Fisher Scientific) and 20 μg of each sample was separated using 4 to 12% polyacrylamide NuPAGE Novex gel (Invitrogen) run at 200 V for 55 min. Semidry transfer (Novex, SD1000) was performed for 45 min at 20 V onto a polyvinylidene difluoride (Immobilon-FL) membrane (EMD Millipore). The membranes were further blocked for 1 h at room temperature using Li-Cor blocking buffer (Li-Cor Biosciences) and incubated overnight with primary antibodies for Lipin-1 (CST #14906), P-STAT6 (CST #56554), STAT6 (CST #5397), and GAPDH (CST #2118). Goat anti-rabbit HRP secondary antibody (Jackson #111-035-144) was added to the membranes and incubated for 2 h at room temperature. Membranes were washed three times with tris buffered saline with tween 20 and incubated in ImmunoCruz Western blotting luminol reagent (Santa Cruz, sc-2048) for 1 min. Images were captured using an Amersham Imager 680 (GE Healthcare Bio-Sciences). Densitometry was performed using IQTL 8.1 (GE Healthcare Biosciences). Bands of interest were normalized to GAPDH.

### Quantitative Real Time PCR

Bone marrow-derived macrophages were treated with IL-4 (R&D Biosystems, 404-ML-050) for 4 h and mRNA was extracted from the cultured cells using RNeasy Mini Kit (Qiagen – 74106) as per manufacturer’s instructions. cDNA template was generated using qScript cDNA SuperMix (Quantabio, 95048). qRT-PCR was performed in a Biorad iCycler with SsoAdvanced Universal SYBER Green SuperMix (Biorad, 172-5271). Primers (Table 1) were obtained from the Harvard primer bank database. Primer specificity was confirmed using primer BLAST and by verifying the presence of a single peak in melt curve analysis. Results were expressed as fold change relative to IL-4 treated WT cells by 2^–ΔΔCt^ method after normalizing with GAPDH.

**TABLE 1:** Primers used for Quantitative Real Time PCR.

*Gapdh*	AGTGGCAAAGTGGAGATT	GTGGAGTCATACTGGAACA
*Arg1*	TGTCCCTAATGACAGCTCCTT	GCATCCACCCAAATGACACAT
*Socs2*	TGCGGATTGAGTACCAAGATGG	CTGTCCGTTTATCCTTGCACA
*Ccl17*	TACCATGAGGTCACTTCAGATGC	GCACTCTCGGCCTACATTGG
*Il10*	CCCATTCCTCGTCACGATCTC	TCAGACTGGTTTGGGATAGGTTT
*Mannr*	GATATGAAGCCATGTACTCC	GGCAGAGGTGCAGTCTGCAT
	TTACTGG	
*Pparg*	GGAAGACCACTCGCATTCCTT	GTAATCAGCAACCATTGGGTCA

### Phagocytosis Assay

Bone marrow-derived macrophages (5 × 10^5^ cells) were cultured on sterile coverslips in culture wells and treated with 20 ng/ml IL-4 for 24 h. Culture medium was then replaced with DMEM, containing pHrodo^TM^ green Zymosan A BioParticles^®^ (Thermo Fisher Scientific, P35365) such that each well receives 0.1 mg zymosan particles. BMDMs were allowed to phagocytose for 1 h under dark incubation and then the assay was stopped by cold PBS wash. Cells seeded on coverslips were then fixed using 4% formaldehyde. Cells on cover slips were washed three times and then stained with DAPI slowfade (Invitrogen, S36938). Immunofluorescent images were taken using Olympus BX51 and evaluated using Image J (1.50a) analysis software. Phagocytic efficiency for each image was calculated by dividing the total number of fluorescent beads by the total number of nuclei in the fluorescent image, thus giving average number of beads per cell. Experiment was performed 3 times with 4 random images per group (*n* = 12).

### Histology

Wound area was carefully excised at 2, 5, and 14 days after wounding and fixed in 10% neutral buffered formalin followed by paraffin embedding. 5 μm thick sections were cut from formalin-fixed paraffin-embedded tissue blocks. Sections were rehydrated, followed by hematoxylin-eosin (H&E) staining and dehydration. Stained sections were then imaged using Olympus BX51. 4× images were compared between each group to assess wound healing. Morphological score of inflammation: Evaluation of cellular infiltrate (polymorphonuclear and mononuclear cells) was done on H&E stained sections using the 10 x objectives. The cells were counted at the wound bed and scored as 0, 1, 2, and 3 (absence of inflammation, Discrete-presence of few inflammatory cells, Moderate-many inflammatory cells and Severe-exaggerated inflammatory cellularity, respectively) for whole skin. The cellularity of the overlying crust or scab was excluded from the score. The scab was made of fibrin and polymorphonuclear cells. The scab was interpreted as either thin (scored as 1) or thick (Scored as 2) based on their morphological appearance on H&E sections. Scoring was performed in a blinded fashion.

### Cytometric Bead Array

Serum cytokine concentration was measured using Biolegend LEGENDplex (Biolegend Mouse inflammation Panel #740446). The assay was performed according to the manufacturer’s instructions, and all samples were run in duplicate. Data was analyzed using the LEGENDplex Data Analysis Software.

### Statistical Analysis

GraphPad Prism 5.0 (La Jolla, CA, United States) was used for statistical analyses. All data was tested for normalcy using the Shapiro Wilks Normalcy test. If data was normally distributed student *T* Test analysis was used for comparison between two data sets. If data was not normally distributed a Mann-Whitney test was performed. All other statistical significance was determined using a one-way ANOVA analysis of variance with a Dunnett’s post-test. All *in vivo* experiments were performed a minimum of two times and all *in vitro* experiments were performed a minimum of three times. Figure legends provide specific details for each data set.

## Results

### Lipin-1 Contributes to IL-4 Elicited Gene Expression

**FIGURE 1:**
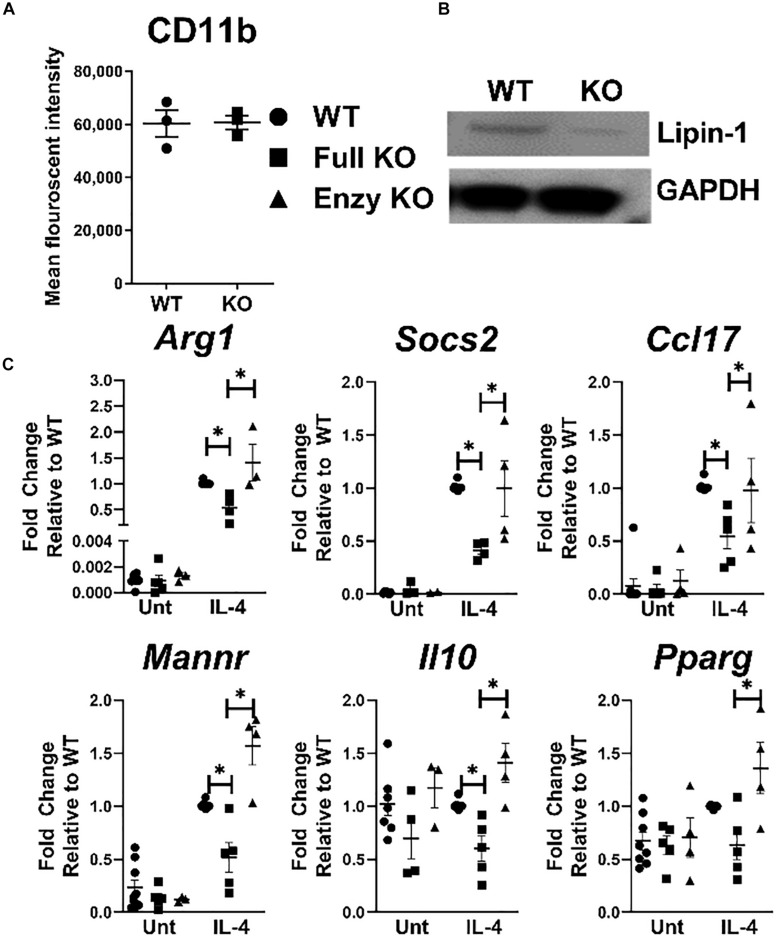
Lipin-1 promotes IL-4 mediated gene expression. **(A)** Flow cytometry was used to quantify CD 11b surface expression of BMDMs from lipin-1^m^KO and littermate controls. Each dot represents an independent experiment. **(B)** Lipin-1 was quantified by Western blot analysis, representative image of three independent experiments shown. **(C)** BMDMs generated from lipin-1^m^KO, lipin-1^mEnzy^KO and their respective littermate control mice. BMDMs were stimulated with 20 ng/ml IL-4 for 4 h. mRNA was isolated and wound healing associated genes were quantified by qRT-PCR. No difference was noted between littermate controls as such they were combined in WT. Each dot represented an individual experiment. Experiments were performed a minimum of three times. All data were normal except for WT IL-4 treated gene expression. Mann Whitney test was used for comparing WT and lipin-1^m^KO; unpaired *T* test used for comparing lipin-1^m^KO and lipin-1mEnzyKO Data presented is mean ± SEM, * *p* < 0.05.

Pro-inflammatory response in macrophages is influenced by lipin-1, but if lipin-1 contributes to wound healing responses by macrophages is unknown. We have previously generated lipin-1^mEnzy^KO mice that express a truncated lipin-1 protein lacking lipin-1 enzymatic activity but retain transcriptional co-regulatory function in myeloid cells (9). Here, we generated lipin-1^m^KO mice that lack the entire lipin-1 protein in myeloid cells. Comparing results between lipin-1^mEnzy^KO mice and lipin-1^m^KO mice allows us to determine the contribution of lipin-1 enzymatic activity and infer the contribution of lipin-1 transcriptional coregulator activity on macrophage function. We have previously demonstrated the ability to generate BMDMs from lipin-1^mEnzy^KO mice and confirmed their phenotype (9). We confirmed that the loss of full lipin-1 did not inhibit BMDM generation based on CD11b staining by flow-cytometry (Figure 1A). Western Blot analysis of proteins collected from cultured BMDMs demonstrated roughly an 85% reduction of lipin-1 protein in lipin-1^m^KO BMDMs, residual lipin-1 protein is due to ineffective Cre excision of lipin-1 (Figure 1B) (26). Having generated macrophages lacking lipin-1, we investigated the contribution of lipin-1 to IL-4 mediated gene expression. BMDMs from lipin-1^mEnzy^KO, lipin-1^m^KO, and appropriate littermate controls were stimulated with 20ng/ml of IL-4 for 4 h. mRNA was isolated and analyzed for the expression of several canonical wound-healing associated genes: *Arg1, Socs2, Ccl17, Mannr, Il10*, and *Pparg* (27). We included littermate controls for both strains; however, no differences were noted between lipin-1^m^KO and lipin-1^mEnzy^KO littermate controls, therefore littermate controls were grouped together as wild type. Expression of several wound healing associated genes in wildtype, lipin-1^mEnzy^KO and lipin-1^m^KO BMDMs (Figure 1C) were comparable. However, IL-4 elicited gene expression was significantly lower in lipin-1^m^KO BMDMs compared to either wild type or lipin-1^mEnzy^KO BMDMs. These results demonstrate that lipin-1 enzymatic activity is dispensable for IL-4 mediated gene expression and suggests that lipin-1 transcriptional co-regulatory activity influences IL-4-mediated gene expression in macrophages.

### Lipin-1 Does Not Influence Surface Expression of IL-4 Receptor or STAT6 Phosphorylation

**FIGURE 2:**
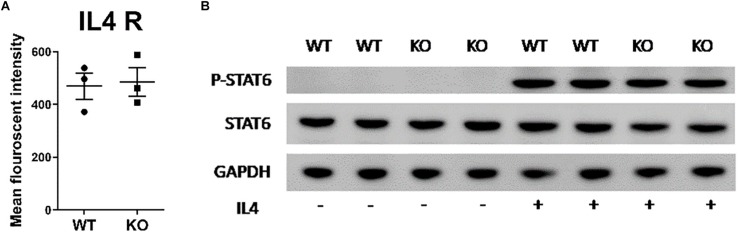
Lipin-1 does not regulate IL-4 mediated STAT6 phosphorylation. **(A)** Flow cytometry was used to quantify surface expression of IL-4R in unstimulated BMDMs from lipin-1^m^KO and litter mate controls (mean ± SEM *n* > 3 from 3 independent experiments). **(B)** BMDMs from lipin-1^m^KO and litter mate controls were stimulated with 20 ng/ml IL-4 for 30 min. Protein was isolated and p-STAT6, STAT6 was quantified by Western blot analysis. A representative blot from three independent experiments is shown.

Lipid membrane composition can influence the localization of receptors and/or signaling through those receptors (28). Lipin-1 is a regulator of glycerol lipid synthesis and the loss of lipin-1 may cause loss of either IL-4 receptor surface expression or signaling through the IL-4 receptor, thus resulting in impaired responses to IL-4. Flow cytometric evaluation of the surface expression of IL-4 receptor showed no difference between wild type and lipin-1^m^KO BMDM (Figure 2A). Ligand binding of the IL-4 receptor-α (IL4Rα) triggers tyrosine phosphorylation at the cytoplasmic tail to facilitate recruitment and subsequent tyrosine phosphorylation of STAT6 by JAK1/JAK3 pathway (29). Wildtype and lipin-1^m^KO BMDMs were stimulated with IL-4 for 30 min (Figure 2B), 1 and 4 h (Supplementary Figure 1) and protein was collected. Total STAT6 and phosphorylated STAT6 was measured by Western blot analysis. Similar levels of STAT6 phosphorylation was observed between wild type and lipin-1^m^KO BMDMs (Figure 2B). These results show that defective IL-4 elicited gene expression in lipin-1^m^KO BMDMs was likely not due to a failure in IL-4 binding to the IL-4 receptor and subsequent STAT6 phosphorylation.

### Lipin-1 Is Required for Phagocytosis

**FIGURE 3:**
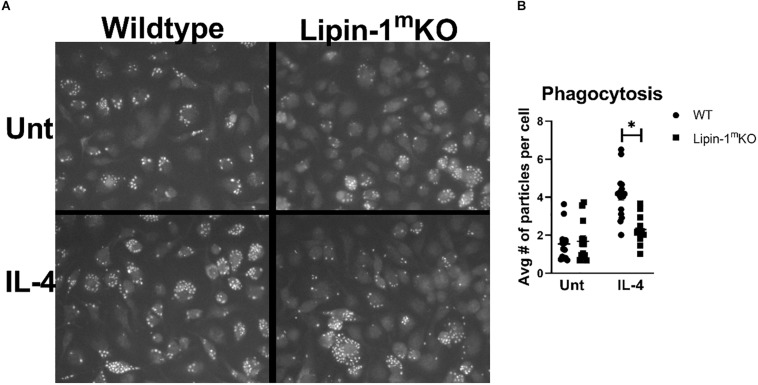
Lipin-1 contributes to IL-4 enhancement of phagocytosis. **(A)** Representative microscopic images of BMDMs from lipin-1^m^KO and littermate control mice fed pHrodo-Green zymosan particles. **(B)** Quantification of number of beads (zymosan beads) divided by number of nuclei in a given image. Experiment was performed 3 times with 4 random image panels taken per group for a total of 12 images. Each dot represents analysis of a single image (mean ± SEM *n* = 12, *p < 0.05).

The reduction in wound healing-associated genes in response to IL-4 suggests that lipin-1 contributes to macrophage wound healing function. Macrophages with a wound healing phenotype can have increased phagocytic capabilities (30). We investigated the ability of BMDMs to phagocytize zymosan beads. We mock treated or IL-4 treated BMDMs from lipin-1^m^KO or litter mate controls for 24 h. We then fed the macrophages pHrodo^TM^ green Zymosan A BioParticles for 1 h. These particles do not fluoresce at 7.6 pH but do fluoresce at acidic pH, making it easier to identify internalized particles. We then imaged using fluorescent microscopy and quantified average number of particles per cell. IL-4 stimulated lipin-1^m^KO BMDMs had fewer particles per cell than wild type BMDMs (Figure 3). These results further implicate the importance of lipin-1 in macrophage function.

### Myeloid-Associated Lipin-1 Contributes to Wound Healing *in vivo*

**FIGURE 4:**
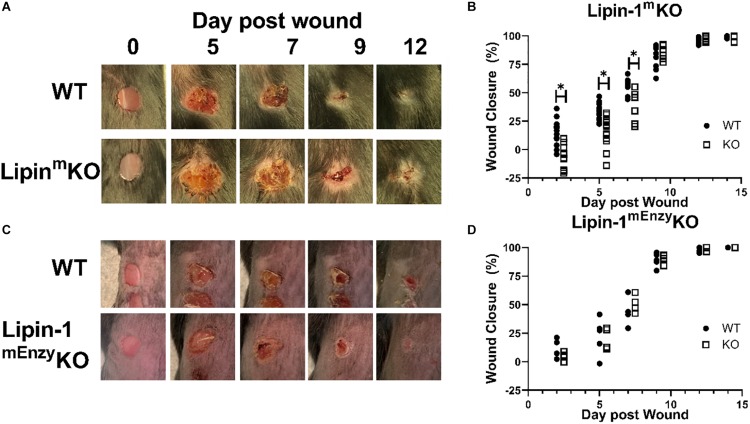
Loss of full lipin-1 delays wound closure. **(A,C)** Representative image of gross lesions. **(B,D)** Percent wound closure as [(area of original wound - area of current wound)/area of original wound] × 100. Wound measurements were made on days 0, 2, 5, 7, 9, 12, and 14 post-wounding. KO mice are shifted by a half day in graph in order to see differences (Experiment was performed a minimum of three times and each dot represents a single animal) *p < 0.05. Each symbol represents an individual mouse.

Our *in vitro* studies suggest that lipin-1 contributes to IL-4 mediated macrophage polarization. We next wanted to determine if these *in vitro* differences contribute to *in vivo* processes as well. Polarization of macrophages to a wound healing phenotype is required for proper wound closure in a full excision wounding model (31, 32). We decided to investigate if the loss of myeloid-associated lipin-1 would alter wound closure. We performed full excision wounding on lipin-1^m^KO, lipin-1^mEnzy^KO and their respective littermate controls. We monitored wound closure at early (day 2 and day 5), middle (day 7 and day 9), and late (day 12 and day 14) stages of wound healing. Lipin-1^m^KO mice had an initial delay in wound healing (days 2, 5, and 7) as compared to litter mate controls (Figures 4A,B). 9 days after wounding, wounds were of comparable size between lipin-1^m^KO mice and litter mate controls. In contrast, lipin-1^mEnzy^KO did not differ from littermate controls in wound healing at any stage of healing (Figures 4C,D). These results demonstrate that lipin-1 enzymatic activity in myeloid cells is dispensable for full excision wound closure and suggests that lipin-1 transcriptional co-regulatory activity in myeloid cells influences full excision wound closure.

### Lipin-1 Deletion Does Not Alter Myeloid Immune Composition

**FIGURE 5:**
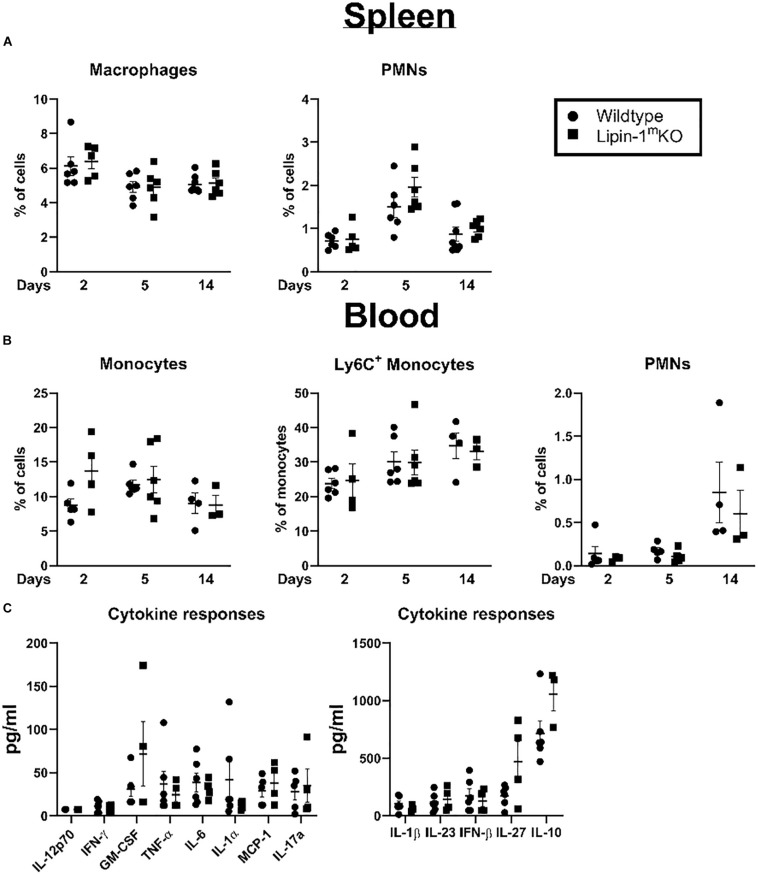
Loss of lipin-1 does not alter systemic immune responses. **(A)** Splenocytes and **(B)** blood cells were stained with a panel of antibodies to quantify monocyte/macrophage and PMN populations. Myeloid populations were defined as CD45^+^, CD3^–^CD19^–^CD11b^+^. PMNs were CD11b^+^ Ly6g^+^ and monocytes were CD11b^+^Ly6G^–^. Each dot represents an individual animal. Experiment was performed twice (mean ± SEM). **(C)** Cytometric bead arrays to quantify cytokine concentrations in serum taken from mice 2 days after wounding Each dot represents an individual animal. Experiment was performed twice (mean ± SEM).

Loss of lipin-1 could potentially influence development of myeloid cells or myeloid mediated systemic responses. We examined myeloid population in the spleen and blood to see if there were any alterations that may explain the delay in wound healing. We isolated the spleen and blood at days 2, 5, and 14 post-wounding. Cells were isolated and stained with a panel of antibodies to quantify macrophages, monocytes, PMNs, and Ly6C^+^ monocytes. We included Ly6C staining as Ly6C^hi^ and Ly6C^lo^ can both contribute to wound healing (33). We observed no significant difference between lipin-1^m^KO and litter mate control mice in any myeloid cell population analyzed in the blood or spleen (Figures 5A,B). In addition to monitoring cellular responses, we also examined mice serum cytokine concentration, 2 days post-wounding. We chose day 2 as this day correlated with the biggest difference in wound size. No differences were noted in serum cytokine responses between lipin-1^m^KO and littermate control mice (Figure 5C). These data suggest that myeloid associated lipin-1 activity that contributes to wound healing is likely mediated in the local environment rather than systemically.

### Loss of Lipin-1 Leads to Alteration in Wound Immune Composition

**FIGURE 6:**
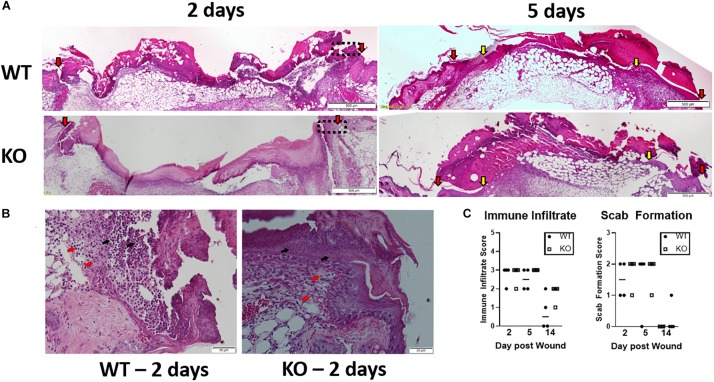
Loss of full lipin-1 delays wound closure. **(A)** H&E depicting epithelial closure of 5mm wounded skin in lipin-1^m^KO and wild type mice post 2 and 5 days of wounding. Red arrow heads indicate host tissue-wound interphase; yellow arrow heads indicate tip of epithelial tongue. **(B)** representative micrographs depicting immune infiltration at the host tissue-wound interphase (highlighted in 5A in rectangular box) post 2 days of wounding. Red arrow points toward monocytes and black arrow points toward neutrophils. **(C)** Pathology score concerning immune cell infiltration and scab thickness. Tissue from 4 mice from each group (*n* = 4). Scale bar **(A)**, 500 μm; **(B)**, 50 μm.

Impaired healing was prominent in the early stage of wound healing. Hence, further histopathological evaluation (Figure 6A) was performed by H&E staining in isolated wounds from lipin-1^m^KO and littermate control mice at 2- and 5-days post wounding. On day 2, slightly interrupted superficial layer with void spaces were seen at the wound site; scattered mononuclear cells and neutrophils were also observed within the superficial layer in control mice. In Lipin-1^m^KO mice, the superficial layer was poorly bridged with large void spaces with more inflammatory cells (Figure 6A). A very thick crest/scab was evident at the wound area which was highly infiltrated with mononuclear cells and neutrophils indicative of hyper inflammatory phase in Lipin-1^m^KO mice. On day 5 epidermal tongue (depicted by yellow arrow heads) extended toward the center of the wound, indicative of wound bridging and healing in control mice. But, in lipin-1^m^KO mice, the crest region was still thick with large number of immune infiltrates and they lacked a definitive epidermal closure and organization, suggestive of impaired healing. Wound closure (interphase between host tissue and wound depicted by red arrow heads) was also improved in the control mice. Scoring of the stained sections (0–3 inflammatory infiltrate and 0–2 crust thickness) by a blinded pathologist showed no significant difference in inflammatory recruitment in lipin-1^m^KO mice (Figures 6B,C).

**FIGURE 7:**
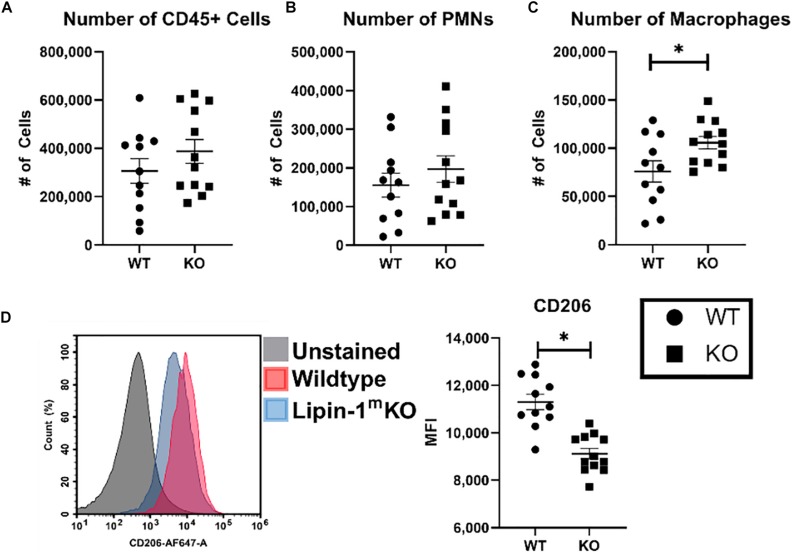
Loss of full lipin-1 leads to reduce CD206 surface expression on macrophages within the wound. Cells were isolated from wounded tissue. Cells were stained with a panel of antibodies to identify **(A)** leukocytes, **(B)** neutrophils, and **(C)** macrophages. We also used an anti-CD206 antibody to characterize macrophage polarization within the wound. **(D)** A representative staining of macrophages from the wound and quantification of mice. Each dot represents an individual wound from 6 animals per group. The experiment was performed twice (*n* = 12) (mean ± SEM). Data was tested for normalcy and *T* test was used for analysis.

We wanted to further investigate whether loss of lipin-1 influence inflammation within the wound during early stage of healing and alter macrophage profiles. To analyze immune cells within the wound we isolated 1 cm^2^ skin including wound and surrounding tissue. Immune cells were isolated from digested skin and then phenotypically characterized by flow cytometry. As we were looking at early time frame within the immune response to wound healing, we concentrated on innate immune cells by quantifying the number of leukocytes (CD45 + cells), number of PMNs (CD45^+^, CD11b^+^, F4/80^–^, and Ly6G^+^), and number of macrophages (CD45^+^, CD11b^+^, and F4/80^+^). Although there was no significant difference in the total number of leukocytes or PMNs within the wounds, we did see a significant increase in the number of macrophages within the wounds of lipin-1^m^KO mice (Figures 7A–C). These observations support our pathological scoring which showed no difference in immune infiltrate. We next examined the surface expression of CD206 (Mannose receptor) on macrophages in the wounds to determine if loss of lipin-1 altered macrophage polarization. We chose CD206 as it is well accepted as a marker for M2 polarization *in vivo* and we had *in vitro* data demonstrating a significant reduction in *MannR* gene expression in lipin-1^m^KO BMDMs (34). Macrophages from wounds of lipin-1^m^KO mice had a significant reduction in surface expression of CD206 compared to WT mice (Figure 7D). These data suggest the importance of lipin-1 in macrophages during wound healing.

## Discussion

Using our lipin-1^m^KO and lipin-1^mEnzy^KO mice, we demonstrated that lipin-1 enzymatic activity is dispensable for wound healing macrophage polarization and provided evidence suggesting that lipin-1 transcriptional co regulator function is required. Only macrophages lacking the entire lipin-1 protein failed to fully express canonical wound healing associated genes in response to IL-4 (5). Furthermore, impaired healing of full excision wound was also observed in lipin-1^m^KO mice but not lipin-1^mEnzy^KO mice. There was no alteration in systemic myeloid immune composition of lipin-1^m^KO mice after wounding, we did observe increased macrophage content and these macrophages had a reduction in the wound healing associated marker CD206. Combined, these data suggest to us that lipin-1 transcriptional co-regulator activity contributes to macrophage wound healing function that promotes wound closure.

Lipin-1 is a multi-functional protein having both enzymatic and transcriptional coregulator function. The removal of exons 3 and 4 of lipin-1 in our lipin-1^mEnzy^KO mice results in truncated lipin-1 that lacks enzymatic activity but retains the ability of lipin-1 to bind to transcription factors such as PPARα and PPARγ (21). Removal of exon 7 from lipin-1 in our lipin-1^m^KO mice causes a missense protein leading to loss of lipin-1 (and both activities) (22). BMDMs from the lipin-1^mEnzy^KO mice had equivalent expression of IL-4 elicited genes as WT BMDMs suggesting that lipin-1 enzymatic activity is dispensable for IL-4 mediated gene expression. Lipin-1^m^KO BMDMs had reduced expression of IL-4 mediated wound healing genes. IL-4 binding to the IL-4R leads to phosphorylation and activation of STAT6 leading to macrophage wound healing polarization. STAT6 binds to DNA promoters that leads to recruitment of PPARγ:RXR transcription factors to promote gene expression in macrophages (2, 29). In adipocytes and hepatocytes, lipin-1 binds to and augments the activity of both PPARα and PPARγ. In addition to augmentation of PPARα and PPARγ activity, lipin-1 inhibits SREBP and NFAT by displacing them from their native promoters (16, 17). We propose lipin-1 transcriptional co-regulator activity promotes macrophages to a wound healing state during IL-4 stimulation. In support of this, macrophages lacking PPARγ fail to polarize to a wound healing phenotype, similar to the phenotype we observed in macrophage lacking full lipin-1 (35). SREBP activity promotes the activation of the NLRP3 inflammasome leading to pro-inflammatory responses (20). While not typically active in macrophages, continued stimulation of macrophages lead to NFAT activity and promotion of pro-inflammatory responses such as IL-6 and TNF-α (19). IL-6 and TNF-α are known to inhibit wound healing polarization. Thus, lipin-1 may also be repressing the activity of NFAT and SREBP allowing wound healing polarization. Our results and these published observations suggest to us that lipin-1 transcriptional coregulator activity promotes wound healing polarization.

Macrophage polarization is critical for effective *in vivo* wound healing where the number and phenotype of the resident and recruited macrophages determine the extent and efficiency of healing (36). Up to 1 day after wounding, pro-inflammatory macrophages initiate an acute inflammatory response; after that time frame, wound healing macrophages promote angiogenesis and tissue formation (37). The loss of enzymatic activity of lipin-1 reduces pro-inflammatory macrophage polarization (7, 9). We observed no defect in wound closure in lipin-1^mEnzy^KO mice compared to litter mate controls demonstrating that lipin-1 enzymatic activity is dispensable for myeloid-mediated wound closure. In contrast, mice lacking both lipin-1 activities had a defect in wound closure. We propose that the lipin-1 transcriptional co-regulatory activity in myeloid cells is responsible for aiding in wound closure during a full excisional wound. Mice lacking PPARγ from myeloid cells (LysMCre model) exhibit a significant delay in wound healing due to compromised granulation, collagen deposition, angiogenesis and a failure in clearance of apoptotic cells (5). PPARγ activation also promotes macrophage associated CD206 expression during a mouse model of liver wounding (38), providing evidence that PPAR activity promotes CD206 expression on macrophages during wounding. We see a significant reduction in CD206 gene expression in lipin-1^m^KO BMDMs and surface expression on macrophages isolated from the wounds of lipin-1^m^KO mice. Myeloid associated SREBP activity also contributes to wound closure, as mice with loss of SREBP activity in myeloid cells (LysMCre model) had enhanced wound closure (18). Lipin-1 can inhibit the activity of SREBP (17). Taking together we propose that the lipin-1 transcriptional co-regulatory activity in myeloid cells promotes beneficial wound closure responses.

We propose that the loss of wound healing observed in our lipin-1^m^KO mice is due to loss of transcriptional co-regulator activity from monocytes and macrophages. However, *LysM*-Cre was used to knockout lipin-1 in our mice, and *LysM* expression is not restricted to monocytes and macrophages. Analysis of *LysM*-Cre–mediated gene deletion demonstrates gene excision in dendritic cells (DC) and neutrophils, as well as monocytes and macrophages (26, 39). We suggest that loss of lipin-1 in DC is not responsible for the difference in wound closure in our lipin-1^m^KO mice. DCs enhance T cell/B cell responses, rather than innate immune responses, and our difference in wound closure is more prevalent in earlier phase of healing (likely prior to T cell responses). The contribution of lipin-1 to DC function is completely unknown and needs to be looked at in the future. Neutrophils clearly contribute to wound healing and we observe PMNs within the wounds of both WT and Lipin-1^m^KO mice (40). Lipin-1 is not readily detected in neutrophils, however, if inflammation drive increases in lipin-1 expression in neutrophils is unknown. Future work will need to address the possibility of neutrophil-associated lipin-1 contribution to wound closure (7). We propose though, that the most likely effect of myeloid loss of lipin-1 is on macrophage function. We observe reduction in wound healing associated gene in lipin-1^m^KO BMDMS that are known to contribute to wound closure, and a reduction in CD206 surface expression on macrophages within the wounds of lipin-^m^KO mice. Thus, though possible loss of lipin-1 in other cells beside macrophages may contribute to reduction in wound closure, at the very least the loss of lipin-1 in macrophages is also a contributing factor.

Our data highlight the role of lipin-1 transcriptional co-regulator activity within macrophage function, specifically for wound healing polarization. Furthermore, we provide evidence that the lack of myeloid-associated lipin-1 transcriptional co-regulator activity has *in vivo* consequences. Macrophage responses are now recognized to play crucial roles in a diverse array of pathologies like atherosclerosis, arthritis, osteoporosis, and sterile inflammation. Beyond sterile inflammation, IL-4 mediated macrophage responses are critical to control and clearance of numerous parasitic infections as well (41). Thus, the contribution of lipn-1 to myeloid cells function is likely to be important beyond sterile inflammation. Future work will be needed to better understand the mechanisms by which lipin-1 transcriptional co-regulator activity drives macrophage function in different pathological conditions of sterile inflammation and parasitic infections.

## Data Availability Statement

The raw data supporting the conclusions of this article will be made available by the authors, without undue reservation, to any qualified researcher.

## Ethics Statement

All animal studies were approved by the LSU Health Sciences Center–Shreveport Institutional Animal Care and Use Committee.

## Author Contributions

SC performed the experimental work, data analysis, and wrote the manuscript. RMS, CB, AY, RM, and RSS assisted with experimental design, experimental work, and data analysis. BF provided critical reagents necessary to complete and finalize experiments as well as intellectual input on the manuscript writing. MW conceived the idea, designed the study, obtained the funding, analyzed and interpreted the data, and wrote and revised the manuscript. All authors were involved in the final approval of the manuscript.

## Conflict of Interest

The authors declare that the research was conducted in the absence of any commercial or financial relationships that could be construed as a potential conflict of interest.
