# Supplementary figures and images for: Lipin-1 Contributes to IL-4 Mediated Macrophage Polarization
Source: Front Immunol. 2020 May 5;11:787. doi: 10.3389/fimmu.2020.00787 (PMC7214697; doi:10.3389/fimmu.2020.00787)

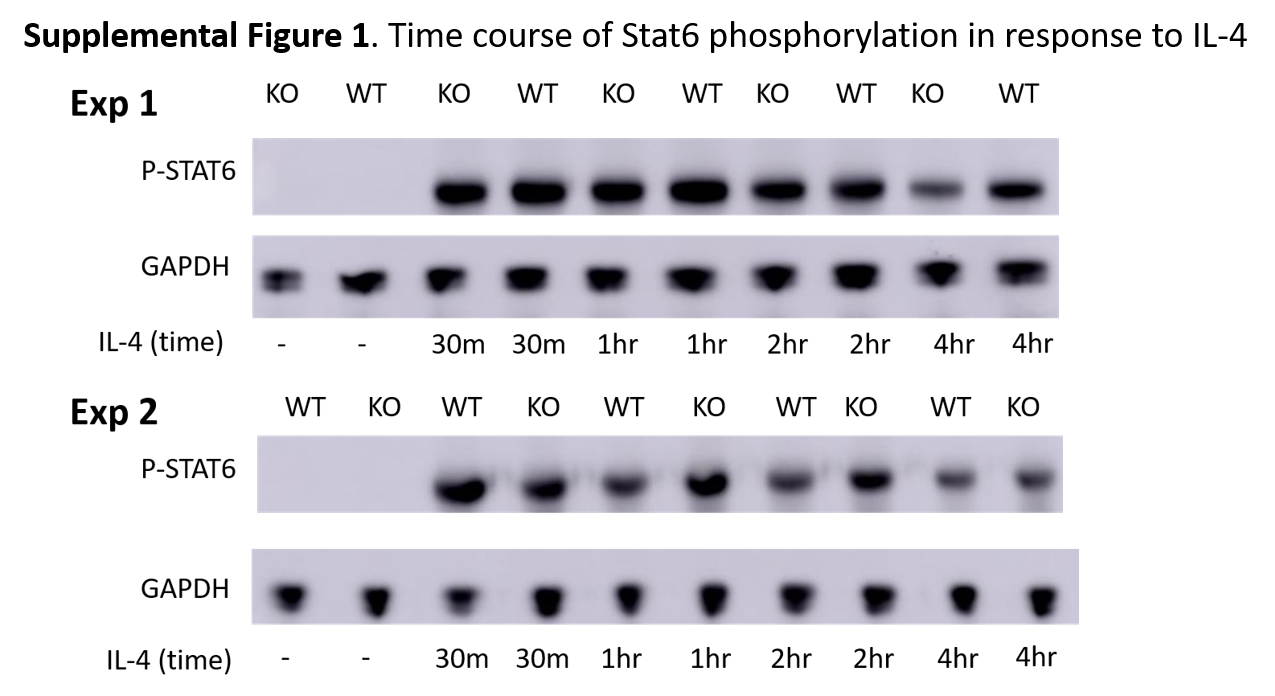

Supplement: Supplementary file 1 [file Image_1.tif]
